# *N*,*N*′-Di­cyclo­hexyl-*N*-(phthaloylglyc­yl)urea

**DOI:** 10.1107/S2414314621006970

**Published:** 2021-07-13

**Authors:** Sylvain Bernès, María Guadalupe Hernández-Linares

**Affiliations:** a Instituto de Física, Benemérita Universidad Autónoma de Puebla, Av. San Claudio y 18 Sur, 72570 Puebla, Pue., Mexico; b Laboratorio de Investigación del Jardín Botánico, Centro de Química, Instituto de Ciencias, Benemérita Universidad Autónoma de Puebla, 72570 Puebla, Pue., Mexico; University of Aberdeen, Scotland

**Keywords:** crystal structure, urea, cyclo­hex­yl, hydrogen bond

## Abstract

The mol­ecular structure of the title compound {systematic name: 1,3-di­cyclo­hexyl-1-[2-(1,3-dioxo-2,3-di­hydro-1*H*-isoindol-2-yl)acet­yl]urea}, C_23_H_29_N_3_O_4_, derived from *N*,*N*′-di­cyclo­hexyl­urea, shows that the tertiary N atom substituted by a cyclo­hexyl and phthaloylglycyl groups adopts a perfectly planar geometry (bond-angle sum = 360.0°). In the same way as for *N*,*N*′-di­cyclo­hexyl­urea, the extended structure of the title compound features N—H⋯O hydrogen bonds, which generate chains of mol­ecules running in the [001] direction.

## Abstract 2

The title urea derivative forms a one-dimensional supra­molecular structure in the solid state, *via* inter­molecular N—H⋯O hydrogen bonds.

## Graphical abstract

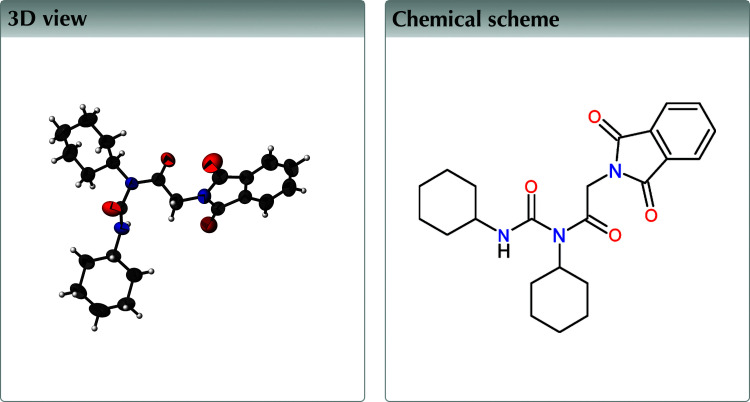

## Structure description

The crystal structure of *N*,*N*′-di­cyclo­hexyl­urea (DCU) was first determined 50 years ago in space group *P*2/*c* (Coiro *et al.*, 1971 ▸), followed by numerous redeterminations, including a wrong claim for a *P* 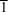 polymorph (Zhu *et al.*, 2009 ▸; the reported triclinic unit cell with *Z*′ = 3 can be restored to the Laue 2/*m* class, using the transformation matrix [1 0 −1/3, 0 0 −1/3, 0 1 −1/3], affording the cell parameters of the actual *P*2/*c* structure with *Z*′ = 1). This cheap compound is an entrance gate for many organic derivatives, through the functionalization of one or two of the amine groups (*e.g*. Orea Flores *et al.*, 2006 ▸; Imhof, 2007 ▸; Pinheiro *et al.*, 2012 ▸). While DCU has been found to be basic enough to coordinate to acid cations such as Nb^5+^ or La^3+^ (Aresta *et al.*, 2010 ▸; Zhang *et al.*, 2016 ▸), its derivatives obtained by functionalization of the amine groups cannot serve as ligands, because of the hindrance between urea substituents.

**Figure 1:**
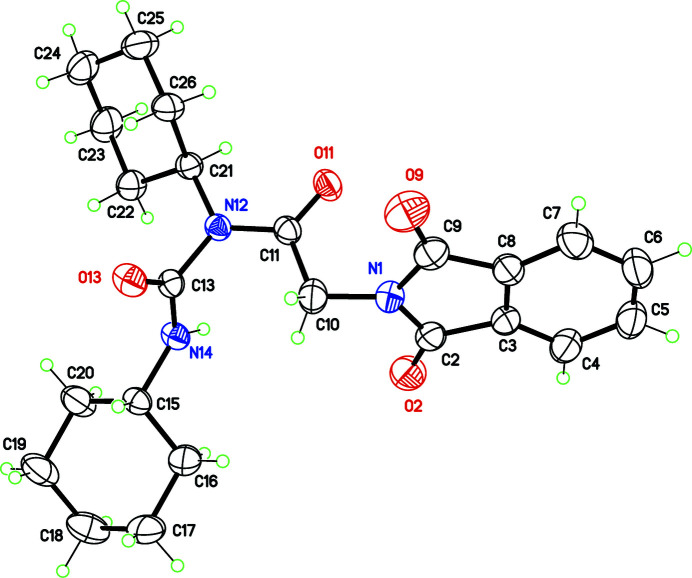
Mol­ecular structure of the title compound, with displacement ellipsoids at the 30% probability level.

In the here-reported structure of the title compound, one amine, N12, is substituted by a phthaloylglycyl group (Fig. 1 ▸). Atom N12 is thus bonded to three bulky groups, and displays a planar geometry: the sum of angles at N12 is exactly 360°. The cyclo­hexyl groups have the normal chair conformation, and the phthaloyl plane is inclined by 46.53 (8) and 44.92 (7)° with respect to the mean planes of the cyclo­hexyl rings. This conformation seems to be suitable for minimizing intra­molecular steric hindrance.

**Figure 2:**
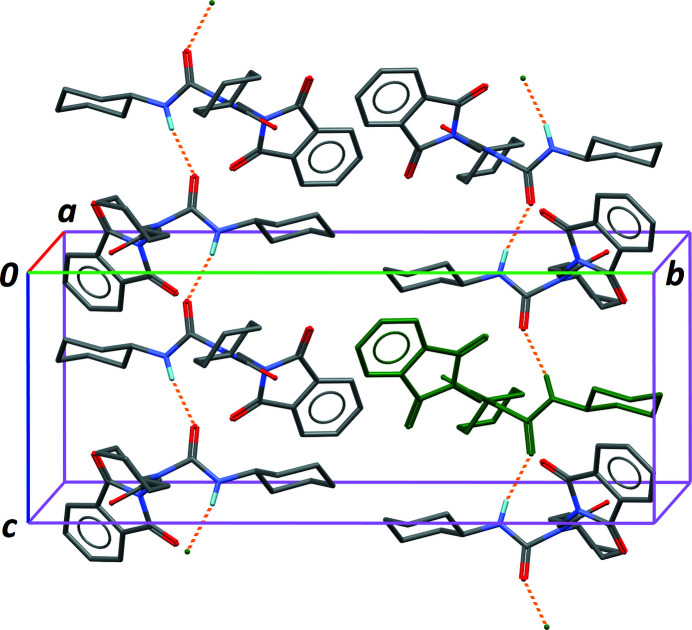
Part of the crystal structure, showing the one-dimensional supra­molecular network formed by N—H⋯O hydrogen bonds (dashed orange bonds, see Table 1 ▸). The green mol­ecule corresponds to the asymmetric unit. All H atoms were omitted, except H14, which is involved in hydrogen bonds.

The non-substituted DCU amine site, N14, is the single available donor group for hydrogen bonding. Weak N—H⋯O hydrogen bonds are formed with the urea carbonyl group as acceptor (Table 1 ▸), forming chains of connected mol­ecules in the crystal, parallel to [001]. Adjacent mol­ecules within this supra­molecular one-dimensional structure are related by the *c* glide planes of space group *P*2_1_/*c*, while the 2_1_ screw axis relates parallel chains in the crystal (Fig. 2 ▸). A consequence of this arrangement is that the phthaloyl aromatic rings inter­act poorly in the crystal: the shortest distance between the centroids of symmetry-related benzene rings is 5.77 Å. The poor packing results in voids of *ca* 30 Å^3^, placed at the unit-cell origin and at the centre of the (**b**, **c**) unit-cell faces. However, these voids seem to be empty, and attempts to model disordered solvent in the crystal did not improve the refinement. The poor crystal packing is reflected in the quite low Kitaigorodskii index of 0.643 (Kitaigorodskii, 1965 ▸; Spek, 2009 ▸).

## Synthesis and crystallization

A solution of 0.5 g (6.66 mmol) of glycine, 0.5 g (3.38 mmol) of phthalic anhydride and 0.224 g of *N,N′*-di­cyclo­hexyl­urea (1 mmol) in 50 ml of glacial acetic acid was maintained under reflux for 30 min. Cooling of this mixture led to separation of a nearly white powder, which was filtered out and washed twice with 10 ml of water, to afford 0.254 g (yield: 62%) of the desired *N,N′-*di­cyclo­hexyl-*N*-(phthaloylglyc­yl)urea derivative. Single crystals were obtained by recrystallization from an EtOAc/hexa­ne/acetone mixed solvent system (7:2:1).

## Refinement

Crystal data, data collection and structure refinement details are summarized in Table 2 ▸.

## Supplementary Material

Crystal structure: contains datablock(s) I. DOI: 10.1107/S2414314621006970/hb4389sup1.cif

Structure factors: contains datablock(s) I. DOI: 10.1107/S2414314621006970/hb4389Isup2.hkl

Supporting information file. DOI: 10.1107/S2414314621006970/hb4389Isup3.cml

CCDC reference: 2094892

Additional supporting information:  crystallographic information; 3D view; checkCIF report

## Figures and Tables

**Table 1:** Hydrogen-bond geometry (Å, °)

*D*—H⋯*A*	*D*—H	H⋯*A*	*D*⋯*A*	*D*—H⋯*A*
N14—H14⋯O13^i^	0.858 (18)	2.031 (19)	2.8741 (16)	167.0 (18)

Symmetry code: (i) 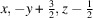 .

**Table 2:** Experimental details

Crystal data	
Chemical formula	C_23_H_29_N_3_O_4_
*M*_r_	411.49
Crystal system, space group	Monoclinic, *P*2_1_/*c*
Temperature (K)	263
*a*, *b*, *c* (Å)	10.2705 (5), 23.5891 (18), 9.3482 (4)
β (°)	97.810 (4)
*V* (Å^3^)	2243.8 (2)
*Z*	4
Radiation type	Ag *K*α, λ = 0.56083 Å
μ (mm^−1^)	0.05
Crystal size (mm)	0.60 × 0.40 × 0.20

Data collection	
Diffractometer	Stoe Stadivari
Absorption correction	Multi-scan (*X-AREA*; Stoe & Cie, 2018 ▸)
*T*_min_, *T*_max_	0.525, 1.000
No. of measured, independent and observed [*I* > 2σ(*I*)] reflections	38438, 4522, 2974
*R*_int_	0.039

Refinement	
*R*[*F* ^2^ > 2σ(*F* ^2^)], *wR*(*F* ^2^), *S*	0.045, 0.133, 1.05
No. of reflections	4522
No. of parameters	275
H-atom treatment	H atoms treated by a mixture of independent and constrained refinement
Δρ_max_, Δρ_min_ (e Å^−3^)	0.16, −0.14

Computer programs: *X-AREA* (Stoe & Cie, 2018 ▸), *SHELXT2014/5* (Sheldrick, 2015*a* ▸), *SHELXL2018/1* (Sheldrick, 2015*b* ▸), *XP* in *SHELXTL-Plus* (Sheldrick, 2008 ▸), *Mercury* (Macrae *et al.*, 2020 ▸) and *publCIF* (Westrip, 2010 ▸).

## full crystallographic data

### Crystal data

**Table:**

C_23_H_29_N_3_O_4_	*F*(000) = 880
*M**_r_* = 411.49	*D*_x_ = 1.218 Mg m^−^^3^
Monoclinic, *P*2_1_/*c*	Ag *K*α radiation, λ = 0.56083 Å
*a* = 10.2705 (5) Å	Cell parameters from 17715 reflections
*b* = 23.5891 (18) Å	θ = 2.3–25.4°
*c* = 9.3482 (4) Å	µ = 0.05 mm^−^^1^
β = 97.810 (4)°	*T* = 263 K
*V* = 2243.8 (2) Å^3^	Prism, colourless
*Z* = 4	0.60 × 0.40 × 0.20 mm

### Data collection

**Table:**

Stoe Stadivari diffractometer	4522 independent reflections
Radiation source: Sealed X-ray tube, Axo Astix-f Microfocus source	2974 reflections with *I* > 2σ(*I*)
Graded multilayer mirror monochromator	*R*_int_ = 0.039
Detector resolution: 5.81 pixels mm^-1^	θ_max_ = 20.5°, θ_min_ = 2.2°
ω scans	*h* = −12→12
Absorption correction: multi-scan (X-AREA; Stoe & Cie, 2018)	*k* = −29→29
*T*_min_ = 0.525, *T*_max_ = 1.000	*l* = −11→11
38438 measured reflections	

### Refinement

**Table:**

Refinement on *F*^2^	Secondary atom site location: difference Fourier map
Least-squares matrix: full	Hydrogen site location: mixed
*R*[*F*^2^ > 2σ(*F*^2^)] = 0.045	H atoms treated by a mixture of independent and constrained refinement
*wR*(*F*^2^) = 0.133	*w* = 1/[σ^2^(*F*_o_^2^) + (0.0664*P*)^2^ + 0.1585*P*] where *P* = (*F*_o_^2^ + 2*F*_c_^2^)/3
*S* = 1.05	(Δ/σ)_max_ = 0.001
4522 reflections	Δρ_max_ = 0.16 e Å^−^^3^
275 parameters	Δρ_min_ = −0.14 e Å^−^^3^
0 restraints	Extinction correction: SHELXL-2018/1 (Sheldrick 2015b), Fc^*^=kFc[1+0.001xFc^2^λ^3^/sin(2θ)]^-1/4^
0 constraints	Extinction coefficient: 0.013 (2)
Primary atom site location: structure-invariant direct methods	

### Special details

**Table:**

Refinement. The refinement was straightforward. All H atoms were placed in idealized positions, except amine H atom (H14), which was found in a difference map, and refined with free coordinates (Table 1).

### Fractional atomic coordinates and isotropic or equivalent isotropic displacement parameters (Å^2^)

**Table:**

	*x*	*y*	*z*	*U*_iso_*/*U*_eq_	
N1	0.50710 (14)	0.65006 (7)	0.53206 (16)	0.0611 (4)	
C2	0.44228 (17)	0.66270 (9)	0.3962 (2)	0.0606 (5)	
O2	0.45849 (15)	0.70516 (7)	0.32921 (18)	0.0883 (5)	
C3	0.35388 (16)	0.61377 (8)	0.35670 (19)	0.0569 (5)	
C4	0.2652 (2)	0.60453 (11)	0.2345 (2)	0.0771 (6)	
H4A	0.251307	0.631325	0.161011	0.093*	
C5	0.1979 (2)	0.55356 (13)	0.2260 (3)	0.0909 (8)	
H5A	0.136821	0.546009	0.145404	0.109*	
C6	0.2194 (2)	0.51398 (12)	0.3340 (4)	0.0947 (8)	
H6A	0.173671	0.479874	0.324236	0.114*	
C7	0.3075 (2)	0.52358 (10)	0.4568 (3)	0.0799 (6)	
H7A	0.321440	0.496745	0.530185	0.096*	
C8	0.37350 (17)	0.57431 (9)	0.46639 (19)	0.0587 (5)	
C9	0.47180 (18)	0.59748 (10)	0.5822 (2)	0.0631 (5)	
O9	0.51337 (16)	0.57772 (8)	0.69790 (15)	0.0910 (5)	
C10	0.60516 (18)	0.68651 (10)	0.6082 (2)	0.0745 (6)	
H10A	0.591711	0.688873	0.708764	0.089*	
H10B	0.596025	0.724342	0.567432	0.089*	
C11	0.74357 (16)	0.66443 (8)	0.59874 (16)	0.0494 (4)	
O11	0.76138 (12)	0.61945 (6)	0.54114 (13)	0.0609 (3)	
N12	0.84226 (13)	0.69750 (6)	0.66247 (12)	0.0442 (3)	
C13	0.81671 (15)	0.75108 (7)	0.72619 (14)	0.0442 (4)	
O13	0.82208 (13)	0.75622 (6)	0.85662 (10)	0.0628 (4)	
N14	0.78851 (14)	0.79217 (6)	0.63139 (13)	0.0486 (4)	
H14	0.7920 (17)	0.7824 (8)	0.544 (2)	0.058*	
C15	0.75042 (16)	0.84949 (8)	0.66761 (15)	0.0489 (4)	
H15A	0.725700	0.848446	0.765210	0.059*	
C16	0.6322 (2)	0.86825 (10)	0.5665 (3)	0.0840 (7)	
H16A	0.560209	0.842203	0.573018	0.101*	
H16B	0.652429	0.867405	0.468148	0.101*	
C17	0.5906 (2)	0.92800 (11)	0.6028 (3)	0.0994 (8)	
H17A	0.516729	0.939667	0.533124	0.119*	
H17B	0.562183	0.927968	0.697566	0.119*	
C18	0.7003 (3)	0.96934 (10)	0.6012 (3)	0.0908 (7)	
H18A	0.672273	1.006388	0.630210	0.109*	
H18B	0.722557	0.972468	0.503966	0.109*	
C19	0.8200 (2)	0.95069 (10)	0.7026 (3)	0.0971 (8)	
H19A	0.800711	0.952286	0.801210	0.117*	
H19B	0.891983	0.976576	0.694241	0.117*	
C20	0.8620 (2)	0.89019 (9)	0.6689 (3)	0.0835 (7)	
H20A	0.893196	0.889700	0.575506	0.100*	
H20B	0.933851	0.878421	0.741015	0.100*	
C21	0.98021 (15)	0.67802 (8)	0.66926 (16)	0.0481 (4)	
H21A	0.985134	0.653999	0.584618	0.058*	
C22	1.07413 (17)	0.72744 (9)	0.6609 (2)	0.0608 (5)	
H22A	1.069335	0.752926	0.741411	0.073*	
H22B	1.048687	0.748397	0.572202	0.073*	
C23	1.21452 (19)	0.70585 (11)	0.6649 (2)	0.0810 (7)	
H23A	1.220726	0.683089	0.579677	0.097*	
H23B	1.273705	0.737837	0.663612	0.097*	
C24	1.2556 (2)	0.67079 (12)	0.7979 (3)	0.0869 (7)	
H24A	1.256342	0.694352	0.883057	0.104*	
H24B	1.343972	0.656574	0.796054	0.104*	
C25	1.1626 (2)	0.62139 (11)	0.8063 (2)	0.0832 (7)	
H25A	1.188750	0.600477	0.894923	0.100*	
H25B	1.168083	0.595950	0.725833	0.100*	
C26	1.02084 (19)	0.64185 (9)	0.80252 (19)	0.0630 (5)	
H26A	0.962661	0.609424	0.801547	0.076*	
H26B	1.013125	0.663887	0.888572	0.076*	

### Atomic displacement parameters (Å^2^)

**Table:**

	*U*^11^	*U*^22^	*U*^33^	*U*^12^	*U*^13^	*U*^23^
N1	0.0527 (8)	0.0596 (11)	0.0689 (9)	−0.0029 (7)	0.0010 (7)	−0.0066 (8)
C2	0.0493 (10)	0.0562 (13)	0.0757 (11)	0.0017 (9)	0.0069 (8)	0.0019 (10)
O2	0.0843 (10)	0.0665 (11)	0.1115 (11)	−0.0071 (8)	0.0036 (8)	0.0236 (9)
C3	0.0472 (9)	0.0567 (13)	0.0664 (10)	0.0015 (8)	0.0069 (8)	−0.0059 (9)
C4	0.0625 (12)	0.0847 (18)	0.0806 (13)	0.0026 (11)	−0.0035 (10)	−0.0108 (12)
C5	0.0634 (13)	0.095 (2)	0.1089 (18)	−0.0025 (13)	−0.0064 (12)	−0.0388 (17)
C6	0.0702 (14)	0.0702 (18)	0.144 (2)	−0.0172 (12)	0.0152 (15)	−0.0283 (17)
C7	0.0726 (14)	0.0632 (16)	0.1071 (16)	−0.0077 (11)	0.0236 (12)	0.0026 (13)
C8	0.0520 (10)	0.0560 (13)	0.0705 (10)	−0.0012 (8)	0.0170 (8)	−0.0047 (9)
C9	0.0577 (11)	0.0713 (15)	0.0615 (10)	0.0056 (9)	0.0127 (8)	−0.0004 (10)
O9	0.0957 (11)	0.1102 (15)	0.0661 (8)	0.0102 (10)	0.0079 (7)	0.0163 (9)
C10	0.0542 (11)	0.0751 (16)	0.0918 (13)	−0.0031 (10)	0.0015 (10)	−0.0281 (12)
C11	0.0571 (10)	0.0467 (11)	0.0442 (7)	−0.0002 (8)	0.0061 (7)	−0.0040 (8)
O11	0.0666 (8)	0.0508 (9)	0.0654 (7)	−0.0037 (6)	0.0089 (6)	−0.0131 (6)
N12	0.0513 (7)	0.0418 (9)	0.0394 (6)	0.0007 (6)	0.0059 (5)	−0.0024 (6)
C13	0.0493 (8)	0.0463 (11)	0.0372 (7)	−0.0017 (7)	0.0067 (6)	−0.0013 (7)
O13	0.0977 (9)	0.0579 (9)	0.0338 (5)	0.0094 (7)	0.0126 (5)	−0.0004 (5)
N14	0.0680 (9)	0.0454 (9)	0.0332 (6)	0.0056 (7)	0.0092 (6)	−0.0012 (6)
C15	0.0610 (10)	0.0452 (11)	0.0416 (7)	0.0043 (8)	0.0112 (7)	−0.0004 (7)
C16	0.0839 (15)	0.0568 (15)	0.1026 (15)	0.0061 (11)	−0.0186 (12)	0.0063 (12)
C17	0.0805 (15)	0.0611 (17)	0.149 (2)	0.0171 (12)	−0.0117 (15)	0.0091 (16)
C18	0.114 (2)	0.0515 (15)	0.1135 (18)	0.0124 (13)	0.0386 (15)	0.0163 (13)
C19	0.0762 (15)	0.0521 (15)	0.168 (2)	−0.0070 (11)	0.0330 (16)	−0.0266 (16)
C20	0.0651 (13)	0.0546 (15)	0.1344 (19)	−0.0020 (10)	0.0267 (12)	−0.0204 (13)
C21	0.0522 (9)	0.0488 (11)	0.0435 (7)	0.0045 (8)	0.0071 (6)	−0.0039 (7)
C22	0.0546 (10)	0.0628 (14)	0.0659 (10)	−0.0028 (9)	0.0111 (8)	0.0024 (9)
C23	0.0540 (11)	0.098 (2)	0.0926 (14)	−0.0021 (11)	0.0140 (10)	−0.0135 (13)
C24	0.0571 (12)	0.102 (2)	0.0968 (15)	0.0174 (13)	−0.0077 (11)	−0.0246 (14)
C25	0.0848 (15)	0.0796 (18)	0.0807 (13)	0.0313 (13)	−0.0056 (11)	−0.0015 (12)
C26	0.0728 (12)	0.0526 (13)	0.0610 (10)	0.0086 (9)	0.0001 (9)	0.0055 (9)

### Geometric parameters (Å, º)

**Table:**

N1—C2	1.384 (2)	C16—C17	1.524 (3)
N1—C9	1.391 (3)	C16—H16A	0.9700
N1—C10	1.437 (2)	C16—H16B	0.9700
C2—O2	1.205 (2)	C17—C18	1.491 (4)
C2—C3	1.484 (3)	C17—H17A	0.9700
C3—C4	1.378 (3)	C17—H17B	0.9700
C3—C8	1.379 (3)	C18—C19	1.512 (4)
C4—C5	1.384 (4)	C18—H18A	0.9700
C4—H4A	0.9300	C18—H18B	0.9700
C5—C6	1.371 (4)	C19—C20	1.536 (3)
C5—H5A	0.9300	C19—H19A	0.9700
C6—C7	1.380 (4)	C19—H19B	0.9700
C6—H6A	0.9300	C20—H20A	0.9700
C7—C8	1.372 (3)	C20—H20B	0.9700
C7—H7A	0.9300	C21—C26	1.521 (2)
C8—C9	1.481 (3)	C21—C22	1.522 (3)
C9—O9	1.202 (2)	C21—H21A	0.9800
C10—C11	1.528 (3)	C22—C23	1.525 (3)
C10—H10A	0.9700	C22—H22A	0.9700
C10—H10B	0.9700	C22—H22B	0.9700
C11—O11	1.215 (2)	C23—C24	1.505 (3)
C11—N12	1.352 (2)	C23—H23A	0.9700
N12—C13	1.436 (2)	C23—H23B	0.9700
N12—C21	1.483 (2)	C24—C25	1.515 (4)
C13—O13	1.2191 (16)	C24—H24A	0.9700
C13—N14	1.319 (2)	C24—H24B	0.9700
N14—C15	1.460 (2)	C25—C26	1.530 (3)
N14—H14	0.858 (18)	C25—H25A	0.9700
C15—C20	1.494 (3)	C25—H25B	0.9700
C15—C16	1.500 (3)	C26—H26A	0.9700
C15—H15A	0.9800	C26—H26B	0.9700
			
C2—N1—C9	112.71 (16)	C16—C17—H17A	109.3
C2—N1—C10	122.89 (19)	C18—C17—H17B	109.3
C9—N1—C10	124.32 (17)	C16—C17—H17B	109.3
O2—C2—N1	124.95 (19)	H17A—C17—H17B	108.0
O2—C2—C3	129.83 (18)	C17—C18—C19	110.8 (2)
N1—C2—C3	105.22 (17)	C17—C18—H18A	109.5
C4—C3—C8	121.53 (19)	C19—C18—H18A	109.5
C4—C3—C2	129.96 (19)	C17—C18—H18B	109.5
C8—C3—C2	108.51 (16)	C19—C18—H18B	109.5
C3—C4—C5	117.0 (2)	H18A—C18—H18B	108.1
C3—C4—H4A	121.5	C18—C19—C20	111.7 (2)
C5—C4—H4A	121.5	C18—C19—H19A	109.3
C6—C5—C4	121.4 (2)	C20—C19—H19A	109.3
C6—C5—H5A	119.3	C18—C19—H19B	109.3
C4—C5—H5A	119.3	C20—C19—H19B	109.3
C5—C6—C7	121.5 (2)	H19A—C19—H19B	107.9
C5—C6—H6A	119.2	C15—C20—C19	111.25 (17)
C7—C6—H6A	119.2	C15—C20—H20A	109.4
C8—C7—C6	117.3 (2)	C19—C20—H20A	109.4
C8—C7—H7A	121.3	C15—C20—H20B	109.4
C6—C7—H7A	121.3	C19—C20—H20B	109.4
C7—C8—C3	121.27 (19)	H20A—C20—H20B	108.0
C7—C8—C9	130.6 (2)	N12—C21—C26	111.30 (13)
C3—C8—C9	108.08 (17)	N12—C21—C22	111.66 (15)
O9—C9—N1	124.9 (2)	C26—C21—C22	111.50 (14)
O9—C9—C8	129.7 (2)	N12—C21—H21A	107.4
N1—C9—C8	105.43 (16)	C26—C21—H21A	107.4
N1—C10—C11	111.23 (17)	C22—C21—H21A	107.4
N1—C10—H10A	109.4	C21—C22—C23	110.29 (18)
C11—C10—H10A	109.4	C21—C22—H22A	109.6
N1—C10—H10B	109.4	C23—C22—H22A	109.6
C11—C10—H10B	109.4	C21—C22—H22B	109.6
H10A—C10—H10B	108.0	C23—C22—H22B	109.6
O11—C11—N12	123.39 (16)	H22A—C22—H22B	108.1
O11—C11—C10	121.39 (16)	C24—C23—C22	111.21 (17)
N12—C11—C10	115.18 (16)	C24—C23—H23A	109.4
C11—N12—C13	121.49 (14)	C22—C23—H23A	109.4
C11—N12—C21	119.66 (14)	C24—C23—H23B	109.4
C13—N12—C21	118.84 (13)	C22—C23—H23B	109.4
O13—C13—N14	125.09 (16)	H23A—C23—H23B	108.0
O13—C13—N12	121.08 (15)	C23—C24—C25	110.95 (18)
N14—C13—N12	113.82 (12)	C23—C24—H24A	109.4
C13—N14—C15	124.48 (12)	C25—C24—H24A	109.4
C13—N14—H14	114.4 (13)	C23—C24—H24B	109.4
C15—N14—H14	121.1 (13)	C25—C24—H24B	109.4
N14—C15—C20	111.57 (15)	H24A—C24—H24B	108.0
N14—C15—C16	110.27 (15)	C24—C25—C26	111.14 (19)
C20—C15—C16	111.69 (18)	C24—C25—H25A	109.4
N14—C15—H15A	107.7	C26—C25—H25A	109.4
C20—C15—H15A	107.7	C24—C25—H25B	109.4
C16—C15—H15A	107.7	C26—C25—H25B	109.4
C15—C16—C17	111.12 (19)	H25A—C25—H25B	108.0
C15—C16—H16A	109.4	C21—C26—C25	110.52 (16)
C17—C16—H16A	109.4	C21—C26—H26A	109.5
C15—C16—H16B	109.4	C25—C26—H26A	109.5
C17—C16—H16B	109.4	C21—C26—H26B	109.5
H16A—C16—H16B	108.0	C25—C26—H26B	109.5
C18—C17—C16	111.6 (2)	H26A—C26—H26B	108.1
C18—C17—H17A	109.3		
			
C9—N1—C2—O2	−178.38 (19)	C10—C11—N12—C13	−3.6 (2)
C10—N1—C2—O2	−1.5 (3)	O11—C11—N12—C21	−2.5 (2)
C9—N1—C2—C3	1.5 (2)	C10—C11—N12—C21	175.45 (15)
C10—N1—C2—C3	178.41 (16)	C11—N12—C13—O13	102.49 (18)
O2—C2—C3—C4	−1.5 (4)	C21—N12—C13—O13	−76.59 (19)
N1—C2—C3—C4	178.66 (19)	C11—N12—C13—N14	−77.72 (18)
O2—C2—C3—C8	177.8 (2)	C21—N12—C13—N14	103.21 (16)
N1—C2—C3—C8	−2.13 (19)	O13—C13—N14—C15	−4.4 (3)
C8—C3—C4—C5	−0.7 (3)	N12—C13—N14—C15	175.80 (14)
C2—C3—C4—C5	178.39 (19)	C13—N14—C15—C20	102.1 (2)
C3—C4—C5—C6	−0.5 (3)	C13—N14—C15—C16	−133.17 (18)
C4—C5—C6—C7	1.1 (4)	N14—C15—C16—C17	179.85 (19)
C5—C6—C7—C8	−0.4 (4)	C20—C15—C16—C17	−55.5 (3)
C6—C7—C8—C3	−0.8 (3)	C15—C16—C17—C18	56.4 (3)
C6—C7—C8—C9	179.4 (2)	C16—C17—C18—C19	−55.8 (3)
C4—C3—C8—C7	1.4 (3)	C17—C18—C19—C20	54.6 (3)
C2—C3—C8—C7	−177.87 (17)	N14—C15—C20—C19	178.28 (19)
C4—C3—C8—C9	−178.77 (17)	C16—C15—C20—C19	54.4 (3)
C2—C3—C8—C9	1.9 (2)	C18—C19—C20—C15	−54.0 (3)
C2—N1—C9—O9	−178.78 (18)	C11—N12—C21—C26	−87.41 (17)
C10—N1—C9—O9	4.4 (3)	C13—N12—C21—C26	91.68 (18)
C2—N1—C9—C8	−0.4 (2)	C11—N12—C21—C22	147.27 (14)
C10—N1—C9—C8	−177.23 (16)	C13—N12—C21—C22	−33.64 (17)
C7—C8—C9—O9	−2.9 (3)	N12—C21—C22—C23	−178.80 (14)
C3—C8—C9—O9	177.28 (19)	C26—C21—C22—C23	55.99 (19)
C7—C8—C9—N1	178.76 (19)	C21—C22—C23—C24	−56.7 (2)
C3—C8—C9—N1	−1.02 (19)	C22—C23—C24—C25	57.2 (3)
C2—N1—C10—C11	−101.7 (2)	C23—C24—C25—C26	−56.5 (2)
C9—N1—C10—C11	74.8 (2)	N12—C21—C26—C25	179.09 (16)
N1—C10—C11—O11	−4.7 (3)	C22—C21—C26—C25	−55.5 (2)
N1—C10—C11—N12	177.35 (16)	C24—C25—C26—C21	55.4 (2)
O11—C11—N12—C13	178.43 (14)		

### Hydrogen-bond geometry (Å, º)

**Table:**

*D*—H···*A*	*D*—H	H···*A*	*D*···*A*	*D*—H···*A*
C10—H10*B*···N14	0.97	2.55	3.114 (3)	117
N14—H14···O13^i^	0.858 (18)	2.031 (19)	2.8741 (16)	167.0 (18)

Symmetry code: (i) *x*, −*y*+3/2, *z*−1/2.
